# Bioengineered Lymphatic Vessels in Synthetic Matrices to Study Breast Cancer Cell Functions

**DOI:** 10.1002/adhm.202503325

**Published:** 2026-01-21

**Authors:** Rodi Odabasi, Lisa A. Krattiger, Magdalini Kanari, Mira A. Jacobs, Lukas O. Moser, Mark W. Tibbitt, Cornelia Halin, Martin Ehrbar

**Affiliations:** ^1^ Department of Obstetrics, University Hospital Zurich University of Zurich Zurich Switzerland; ^2^ Department Research University Center for Dental Medicine Basel UZB University of Basel Basel Switzerland; ^3^ University Children's Hospital Zurich Zurich Switzerland; ^4^ Macromolecular Engineering Laboratory Department of Mechanical and Process Engineering ETH Zurich Zürich Switzerland; ^5^ Institute of Pharmaceutical Sciences Department of Chemistry and Applied Biosciences ETH Zurich Zurich Switzerland

**Keywords:** Breast cancers, Co‐cultures, In vitro models, Lymphatic vessels, Synthetic hydrogels

## Abstract

Lymphatic vessels play an important role in pathologies, such as cardiovascular diseases or metastasizing cancers. However, mechanisms involved in cancer metastasis through lymphatic vessels remain largely unknown. In this study, we engineer 3D lymphatic microvessels by co‐culturing human lymphatic endothelial cells (hLECs) with human bone marrow‐derived mesenchymal stromal cells (hBM‐MSCs) in synthetic and fully defined enzymatically crosslinked poly(ethylene glycol) (TG‐PEG) hydrogels. By tuning the stiffness of the TG‐PEG scaffolds, varying seeding densities of hLECs and hBM‐MSCs, and stimulation with lymphangiogenic factors, we identify suitable culture conditions for lymphatic network formation. Immunocytochemistry‐based evaluations reveal the formation of lymphatic structures consisting of hLECs that are ensheathed with basement membrane extracellular matrix components and hBM‐MSCs, which express perivascular‐specific markers. Finally, the addition of non‐invasive MCF‐7 and invasive MDA‐MB‐231 breast cancer cells to the forming lymphatic structures shows a clear negative influence of cancer cells on the length of lymphatic networks. Moreover, we observe a closer association of invasive than of non‐invasive cancer cells with the lymphatic networks, which can be inhibited by blocking of CCL21. Our findings present a promising approach to investigate the cancer microenvironment, as our model can serve as a tool to study physiological lymphangiogenesis and pathological phenomena in vitro.

## Graphical abstract

Lymphatic vessels are involved in cancer metastasis. To study the interplay between metastasizing cancer cells and lymphatic vessels under highly reproducible conditions, advanced in vitro models are required. In this work, 3D lymphatic networks are formed in biomimetic hydrogels and their interactions with invasive and non‐invasive cancer cell‐lines are studied.

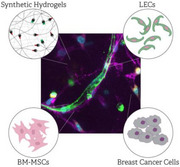

## 1 Introduction

Malignant tumors and their metastases are among the leading causes of death in the industrialized world [1, 2, 3]. As one of the possible routes of tumor cell metastasis, the lymphatic system has recently attracted attention as a potential target for new anti‐metastatic treatments [4, 5]. Physiologically, the lymphatic vasculature plays an important role in draining interstitial fluid and proteins, and transporting dietary fats from the intestine [6, 7]. Additionally, the lymphatic vasculature exercises an indispensable role in transporting immune cells and resolving inflammation, which is essential for tissue regeneration and repair in vivo [8]. These functions are enabled by the specialized architecture of the lymphatic network. The initial lymphatics (lymphatic capillaries) consist of a layer of lymphatic endothelial cells (LECs) and are devoid of any supporting cells [9]. LECs form the innermost layer and act as a barrier between the lymph and the surrounding tissue [10]. To facilitate the uptake of fluid and cells, lymphatic capillaries have an incomplete basement membrane and lack tight cell‐to‐cell junctions [11]. In contrast, LECs in downstream collecting vessels are connected by tight cell‐to‐cell junctions. Moreover, collectors contain valves that are associated with contractile lymphatic muscle cells to support the directed lymph flow to the proximal lymph nodes [12]. In pathological situations, such as cancer, the lymphatic system is thought to be tightly intertwined with the metastasis of cancer cells [12, 13]. Some carcinomas, for example primary breast cancers, are documented to preferentially metastasize through the lymphatic vasculature [14]. Therefore, understanding the early mechanisms of metastasis, such as the intravasation of cancer cells into the lymphatic capillaries and collecting vessels would be highly desirable. However, the formation and the possible remodeling of lymphatic capillaries and collecting vessels in the cancer microenvironment remains insufficiently understood [13, 15].

Moreover, to study lymphatic vessels and their interaction with cancer cells under defined conditions, in vitro engineered 3D lymphatic vessels, which resemble their native counterparts, would be desirable [16, 17]. While several elegant models of 3D lymphatic vessels have been described, the majority of work in the field still focuses on models of blood vessels [18, 19]. These engineered 3D lymphatic vessels were cultured in naturally derived, synthetic and hybrid hydrogels. For example, Marino et al. engineered skin grafts with functional blood and lymphatic capillaries in fibrin hydrogels [20]. Landau et al. used a collagen‐based scaffold to engineer functional lymphatic vessel networks [21]. In both models, the functionality of the engineered vessels was shown by their anastomosis with the host tissue when transplanted into mice. Lee et al. engineered a lymphatic tumor 3D microenvironment in fibrin hydrogels using a microfluidic chip [22]. They showed morphogenic differences of the lymphatic networks depending on the type of cancer present. Gibot et al. established lymphatic 3D reconstruction in a scaffold‐free approach and investigated the effects of VEGFR‐3 blocking [23, 24]. Henderson et al. used a microfluidic chip to bioengineer lymphatic vessels to test vessel structure and barrier function [25]. Ilan et al. expanded on this by investigating the role of VEGF‐A/C on sprouting and junction formation [26]. With the exception of the study by Henderson et al. and Ilan et al., different types of stromal cells were used to support the formation of the lymphatic networks. These early lymphatic models show promising properties. However, they are predominantly based on naturally occurring biomaterials, which are stunted by their insufficient stability and their inherent biological signals.

Various biomaterials with defined and tunable properties, such as alginate, hyaluronic acid (HA) and poly(ethylene glycol) PEG‐based hydrogels have been developed and used in the past decade for the engineering and regeneration of tissues [27]. Due to their modular design, these hydrogels can be tailored for specific needs by the incorporation of defined sites for cell adhesion and proteolytic degradation as well as the adjustment of mechanical properties [27, 28, 29]. For example, Hooks et al. used synthetic hydrogel scaffolds to investigate sprouting of explanted lymphatic collecting vessel segments [30]. Alderfer et al. used HA hydrogels to engineer 3D lymphatic structures without the use of support cells [31]. We have recently used transglutaminase (factor XIIIa) cross‐linked PEG (TG‐PEG) hydrogels to engineer blood vessel micro‐capillaries. This was achieved by the co‐encapsulation of human bone marrow‐derived mesenchymal stromal cells (hBM‐MSCs) and human umbilical vein endothelial cells (HUVECs) [32]. The study revealed that the presence of cell adhesion sites, the ability for proteolytic remodeling and the substrate stiffness were all essential parameters for microvessel formation in these synthetic matrices. The stimulation with angiogenic factors such as vascular endothelial growth factor (VEGF) and fibroblast growth factor (FGF) enhanced this process. During the formation of blood microvessels, the secretion of extracellular matrix (ECM) components and paracrine signals by stromal support cells was critical in the synthetic TG‐PEG hydrogels [27, 32, 33]. Despite the substantial advances with synthetic hydrogel‐based 3D blood vessel models to study interactions with cancer cells, currently, similar models have not been described for lymphatic vessels.

In this study, we engineered 3D lymphatic vessel‐like structures in TG‐PEG hydrogels by co‐encapsulation of juvenile or adult dermal hLECs and hBM‐MSCs. We investigated effects on lymphatic network formation depending on TG‐PEG stiffness, cell densities and cell type ratios, and growth factor stimulation. The structures were morphologically characterized for lymphatic‐, vascular support‐ and basement membrane‐specific markers. Next, we investigated the applicability of our model in a disease context. To mimic a cancer microenvironment, we studied the formation of lymphatic networks in the presence of different densities of non‐invasive and invasive breast cancer cell lines MCF‐7 and MDA‐MB‐231, respectively. Next, we investigated a potential mechanism behind the differential interaction of the cancer cell lines with the lymphatic vessels. Therefore, we investigated the role of C‐C motif chemokine Ligand 21 (CCL21) on the interaction of the studied breast cancer cell lines with the lymphatic microvasculature.

## 2 Results and Discussion

### 2.1 TG‐PEG polymer Content for Culture

**FIGURE 1:**
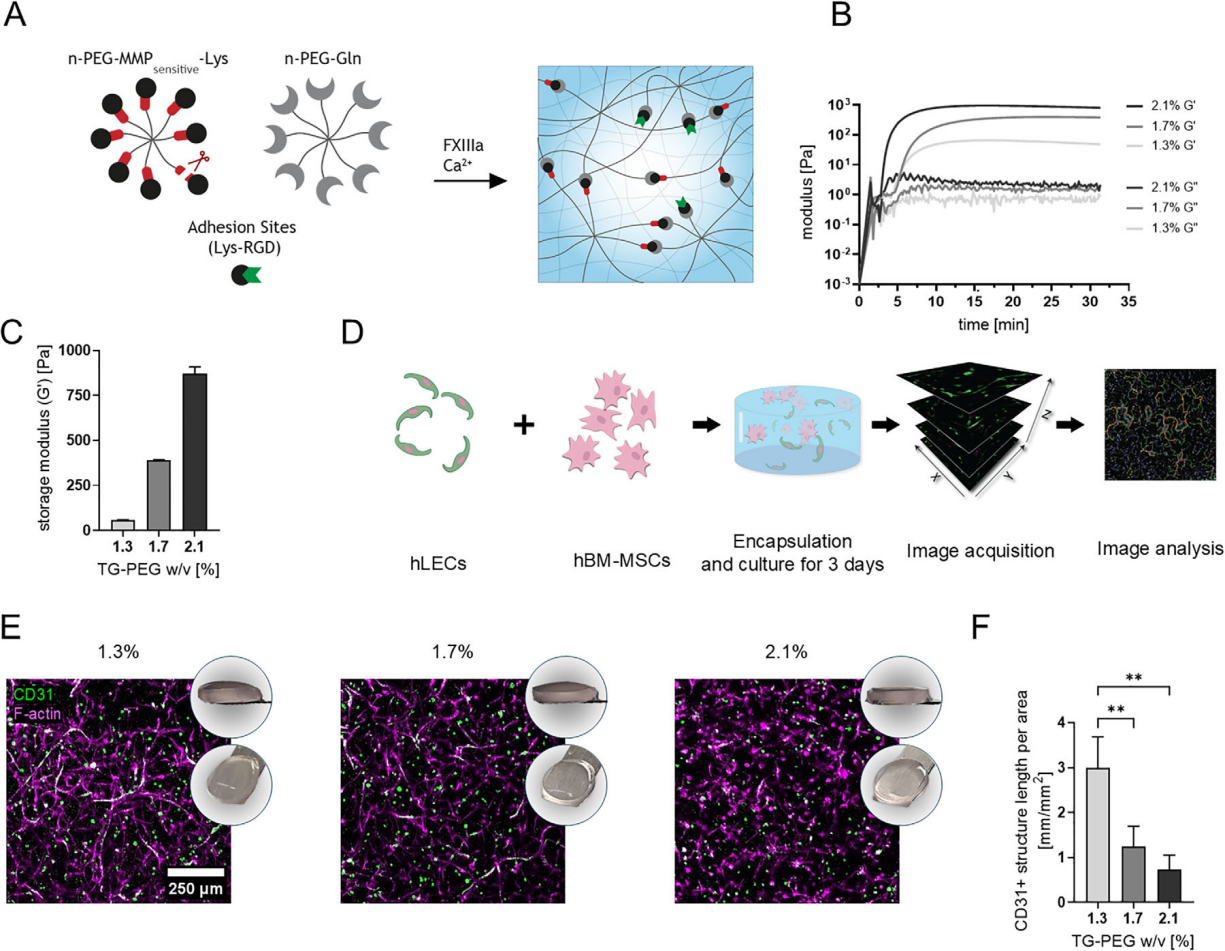
TG‐PEG characterization and CD31 network assessment related to TG‐PEG stiffness. A) Schematic of crosslinking of PEG polymers with adhesion ligand RGD by Factor XIIIa. MMP‐cleavable sites enable cellular remodeling of the formed hydrogels; B) Time‐sweep of storage and loss moduli of different weight percentages (w/v) of TG‐PEG hydrogels, representative measurements (n≥4); C) Final storage moduli of different TG‐PEG formulations (w/v; n≥4); D) Schematic of the workflow for fabricating cell‐laden hydrogels and of the image‐based analysis of the CD31‐positive networks; E) Qualitative confocal images consisting of maximum intensity projections of stacks (total height 210 µm, z‐step 10 µm) show CD31‐positive structures in green and F‐actin‐positive structures in magenta. Also depicted are digital camera photographs of the assessed TG‐PEG hydrogels (w/v); F) Quantification of total length of CD31‐positive structures from images similar to as shown in E, n≥4). Ordinary one‐way ANOVA with Tukey‐Kramer test; **p < 0.01.

The hydrogel backbone consisted of 8‐arm PEG‐VS precursors functionalized with MMP‐cleavable sites as well as peptide sequences for the enzymatic crosslinking via transglutaminase FXIIIa, as previously characterized [28]. To foster cell adhesion, Lys‐RGD was added at 50 µM, which had previously been shown to be sufficient for the spreading of hBM‐MSCs [29]. To characterize the effect of polymer content on gelation kinetics and mechanical properties, rheological tests were performed. A time‐sweep at 4% strain and 1 Hz showed crosslinking to occur between 2 and 5 min and complete gelation (stabilization to a plateau modulus) after ≈10, 15 and 20 min for 2.1%, 1.7%, and 1.3% w/v, respectively (Figure 1B). Final storage moduli (G’) as recorded after 30 min of addition of FXIIIa were 70 ± 15 Pa for 1.3%, 388 ± 12 Pa for 1.7% and 862 ± 60 Pa for 2.1% w/v TG‐PEG (Figure 1C). Moreover, volumetric swelling of the hydrogels was assessed by weighing 15 µL hydrogels before and after 24 h incubation in culture medium. While swelling was minimal (0.85–1.08), a slight trend of increasing swelling with increasing polymer content was observed (Figure S1A).

Next, to test if elongated lymphatic structures can form in TG‐PEG hydrogels, conditions previously established for co‐cultures of hBM‐MSCs and HUVECs were employed as a starting point [32, 34]. Therefore, hLECs were co‐encapsulated at 10^6^ cells/mL with an equal amount of hBM‐MSCs in 15 µL TG‐PEG hydrogels at different weight percentages. The hydrogels were cultured with growth factor supplementation of 50 ng/mL FGF‐2 for 3 days before staining, imaging and image‐based analysis (Figure 1D). After 3 days of culture, elongated lymphatic structures and an underlying dense network of supporting hBM‐MSCs could be visualized by confocal imaging of CD31 and F‐actin‐stained hydrogels (Figure 1E). With image‐based analysis using Angiogenesis Analyzer for ImageJ [35], the total network length of the CD31‐positive structures was quantified. The pan‐endothelial marker CD31 was chosen, as not all lymphatic cells stained positive for the lymphatic‐specific maker LYVE‐1 (Figure S1B). Analysis of the CD31‐positive structures confirmed that with decreasing polymer content and, consequently, lower hydrogel stiffness, the formation of lymphatic networks was more efficient (Figure 1F). The same trend could be qualitatively assessed in the F‐actin‐positive structures. These findings are consistent with previous observations for co‐cultures of hBM‐MSCs and HUVECs [32]. These findings can be explained, as with higher TG‐PEG concentration there are more polymer crosslinks that need to be degraded for cells to spread and migrate. It could be shown that pore sizes are only minimally affected by TG‐PEG concentrations, with pore sizes ranging between 10–50 nm [36]. Finally, for downstream evaluations, the intermediate TG‐PEG concentration of 1.7% was selected as a potentially sensitive condition to determine both enhanced or reduced lymphatic growth.

### 2.2 Influence of Co‐Culture Composition and Growth Factor Supplementation on Lymphatic Vessel Formation

**FIGURE 2:**
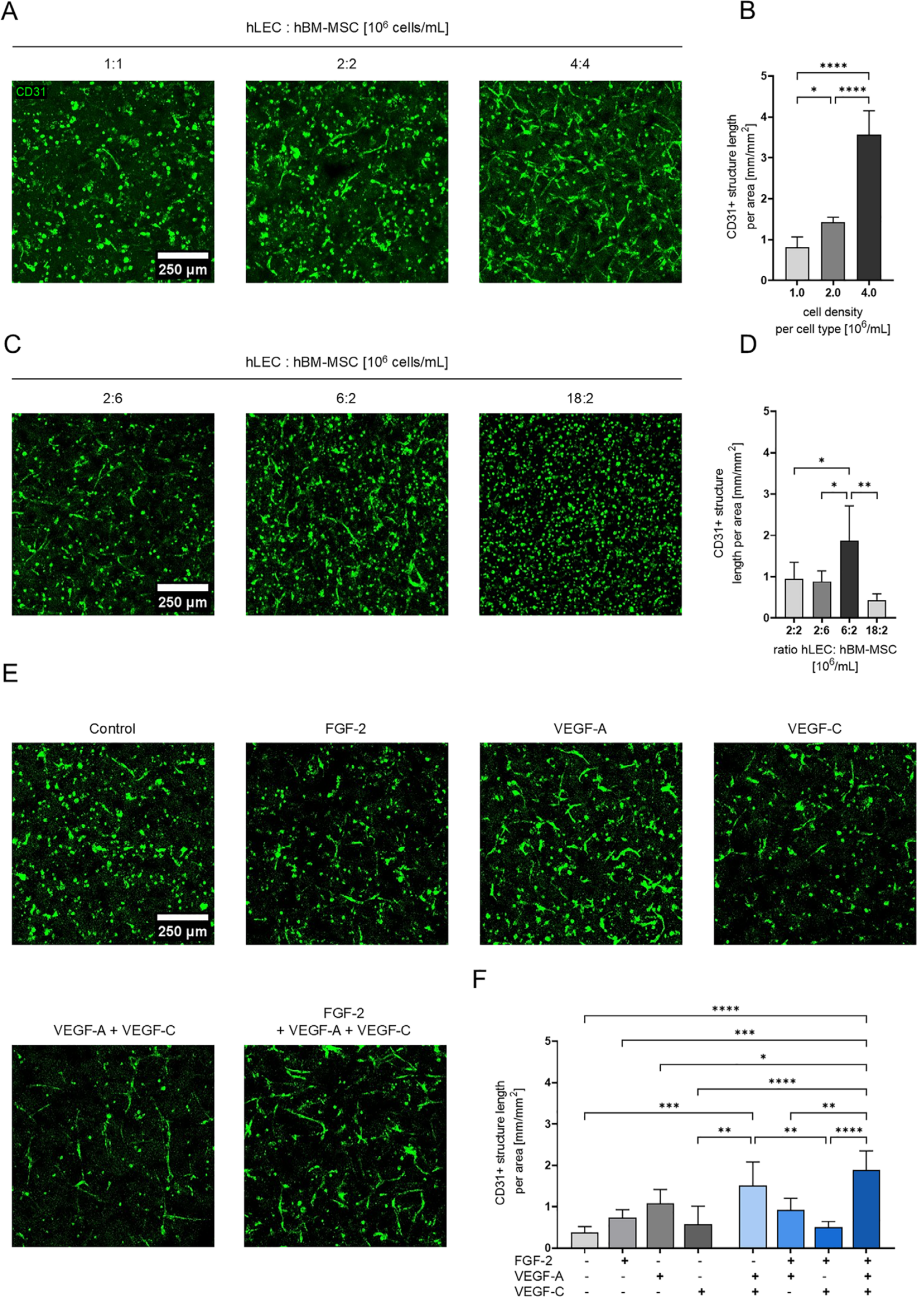
Impact of co‐culture composition and growth factor supplementation on hLEC structures in 1.7% TG‐PEG hydrogels. A) Representative confocal images of different cell densities, numbers indicating the density in 10^6^ cells/mL of both hLECs and hBM‐MSCs (maximum intensity projections of confocal stacks with a total height of 210 µm, z‐step 10 µm, CD31 signal in green); B) Quantification of total length of CD31‐positive structures from images similar to those shown in A; n≥4. Ordinary one‐way ANOVA with Tukey‐Kramer test; *p < 0.05, ****p < 0.0001. C) Representative confocal images of different cell type ratios, numbers indicating the density of both hLECs and hBM‐MSCs (maximum intensity projections of confocal stacks with a total height of 210 µm, z‐step 10 µm) showing CD31‐positive structures in green; D) Quantification of total length of CD31‐positive structures from images similar to those shown in C (n≥4). Ordinary one‐way ANOVA with Tukey‐Kramer test; *p < 0.05, **p < 0.01; E) Representative confocal images (maximum intensity projections, total height 210 µm, z‐step 10 µm, CD31 signal in green) of control condition (cell culture basal medium without growth factor) and cell culture basal medium supplemented with growth factors. The growth factors were supplemented at concentrations of 50 ng/mL (both for single and combined supplementation); F) Quantification of total length of CD31‐positive structures from images similar to those shown in E (n≥4). Ordinary one‐way ANOVA with Tukey‐Kramer test; *p < 0.05, **p < 0.01, ***p < 0.0001, ****p < 0.0001.

Cell seeding density and ratio of endothelial cells and supportive cells have been described to influence the formation of 3D blood vessel networks [33, 34]. The findings for blood endothelial cells (BECs) were re‐assessed (Figure S2A). Next, the influence of cell concentrations on lymphatic network length was evaluated by establishing co‐cultures of 1, 2 and 4×10^6^ cells/mL of each cell type (Figure 2A). Image‐based quantifications revealed that with higher cell densities, significantly longer structures were formed (Figure 2B). To assess the effect of cell type ratios on lymphatic vessel formation, the intermediate seeding density of 2 × 10^6^ cells /ml was chosen for one cell type, while the seeding density of the other cell type was increased. Cell densities of 2×10^6^/mL both hLECs and hBM‐MSCs, 2×10^6^/mL hLECs and 6×10^6^/mL hBM‐MSCs, 6×10^6^/mL hLECs and 2×10^6^/mL hBM‐MSCs, and 18×10^6^/mL hLECs and 2×10^6^/mL hBM‐MSCs were compared (Figure 2C). Significantly more lymphatic structures formed in the condition with 6:2 ×10^6^/mL hLECs:hBM‐MSCs (Figure 2D). Interestingly, structure formation was limited at the highest ratio of 18:2 ×10^6^/mL hLEC:hBM‐MSC, as well as in the absence of hBM‐MSCs (Figure S2B), which is in line with previous observations [21, 22]. Moreover, qualitative assessment of the supporting cells (as visualized by staining for F‐actin; Figure S2C, D) suggests a minor effect of the co‐culture composition for these cells except for the 18:2 ×10^6^/ mL hLEC:hBM‐MSC ratio where the supporting cells appeared not to be able to spread. A possible explanation could be the nutrient competition resulting from the overall high cell density. In short, the encapsulation of balanced ratios of hLECs and hBM‐MSCs led to the formation of lymphatic vessel structures in TG‐PEG hydrogels.

Until this point, all cultures had been grown in the presence of 50 ng/mL of FGF‐2. However, the induction of lymphatic vessel growth has been described to depend on the presence of VEGF‐C [6, 37]. Therefore, to assess the pro‐lymphangiogenic role of selected relevant growth factors, hLECs and hBM‐MSCs were co‐encapsulated at 2×10^6^ cells/mL each in 1.7% TG‐PEG matrices. As a control, cultures were grown in the complete absence of growth factors. Growth factor treatments consisted of 50 ng/mL FGF‐2, 50 ng/mL VEGF‐A, 50 ng/mL VEGF‐C or combinations thereof (Figure 2E,F). When comparing single growth factor supplementation, the most elongated structures formed when supplemented with VEGF‐A. When cultures received growth factor combinations, the combination of 50 ng/mL of each FGF‐2, VEGF‐A and VEGF‐C yielded the longest lymphatic networks, both when evaluated in maximum projections (Figure 2F) or in 3D assessment (Figure S3B). Qualitative assessment of the supporting cells (as visualized by staining for F‐actin; Figure S3A) suggests a stimulatory effect of the growth factors on the supporting cells, enhancing their maintenance and spreading within the 3D cultures. Interestingly, BECs showed the same result of highest network length when cultured with supplementation of the combination of FGF‐2, VEGF‐A and VEGF‐C (Figure S2A). While VEGF‐A is typically associated with angiogenesis and VEGF‐C with lymphangiogenesis, these two isoforms exhibit overlapping VEGF‐receptor binding properties [37, 38], which may explain our observations. Notably, others have previously reported the combined use of VEGF‐A and VEGF‐C, however, direct comparisons to their results are challenging as most studies used a base culture medium such as EGM‐2, which is supplemented with various growth factors [20, 21, 22, 24]. In our setup, when co‐cultures received a combination of FGF‐2, VEGF‐A and VEGF‐C, the longest lymphatic networks formed, pointing to their additive function during this morphogenetic process. Moreover, after combined treatment with FGF‐2, VEGF‐A and VEGF‐C significantly more Prox‐1‐ and LYVE‐1‐positive cells could be detected in elongated vessel‐like structures (Figure S4A). Taken together, these findings indicate that co‐cultures of hLECs and hBM‐MSCs supplemented with a combination of pro‐lymphangiogenic and pro‐angiogenic factors can be used to engineer 3D lymphatic vessel structures in synthetic TG‐PEG matrices.

### 2.3 Lymphatic Vessel Morphology

**FIGURE 3:**
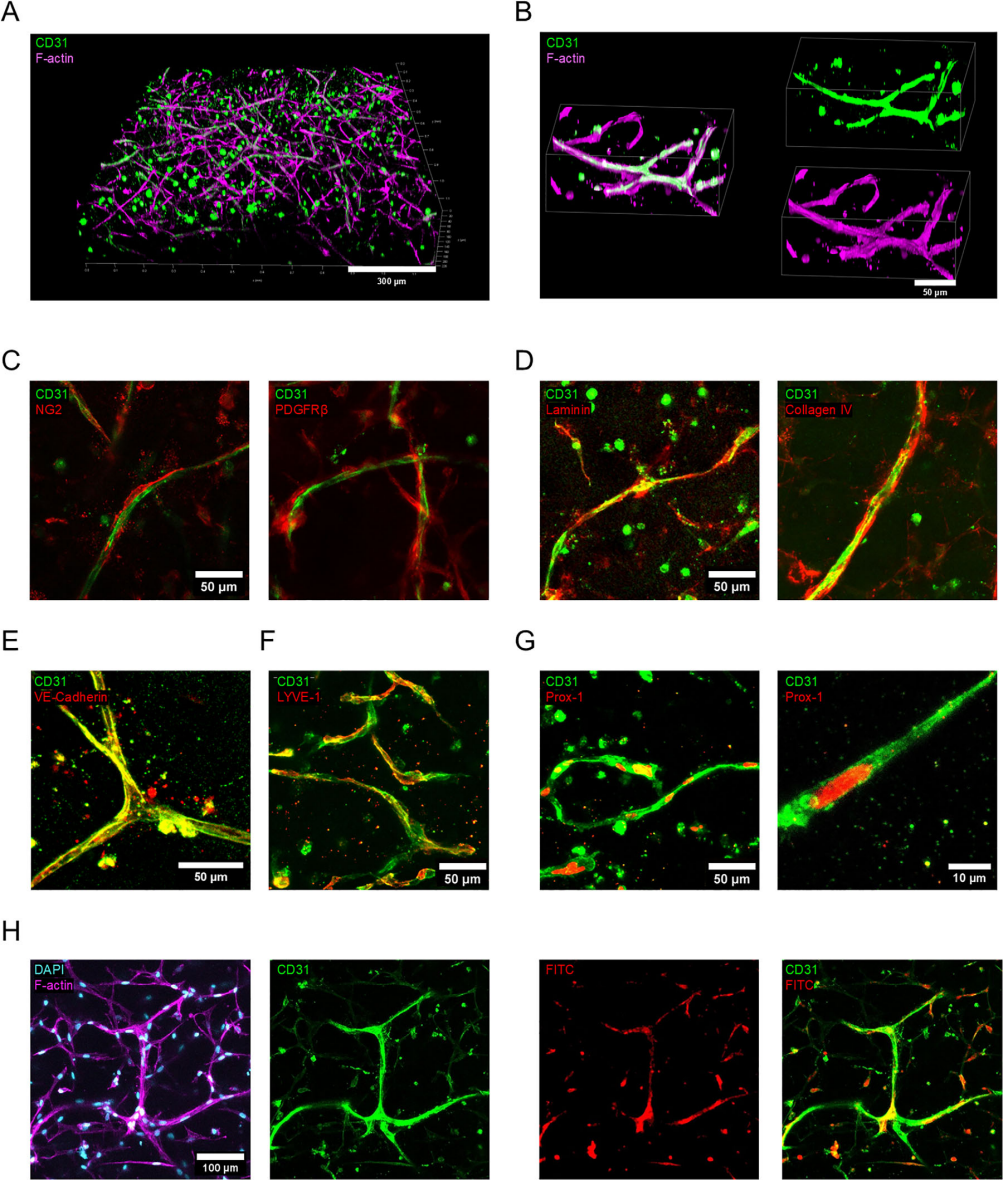
Characterization of hLEC tube‐like structures. A) Image depicts 3D reconstruction of confocal stack showing CD31‐positive structures in green and F‐actin structures in magenta (total height 223.2 µm, z‐step 2.4 µm); B) Higher magnification images depict 3D reconstructions from confocal stacks (total height 101 µm, z‐step 0.9 µm); C) Confocal images of maximum projection of perivascular cell markers NG2 and PDGFRβ (red) with CD31‐positive structures (green) (total height 50.4 µm, z‐step 2.4 µm); D) Maximum projection of confocal images show basement membrane markers laminin and collagen IV (red) and CD31‐positive structures (green) (total height 103.2 µm, z‐step 2.4 µm); E) Confocal images of maximum projection show VE‐Cadherin (red) in overlay with CD31‐positive signal (total height 76.8 µm, z‐step 2.4 µm); F) Lymphatic capillary‐specific marker LYVE‐1 (red) could be observed on some, but not all CD‐31‐positive (green) structures (total height 112.2 µm, z‐step 5.1 µm); G) The lymphatic‐specific marker Prox‐1 (red) could be observed in nuclei residing inside CD31‐positive (green) structures (total height 98.4 µm, z‐step 2.4 µm; left). Higher magnification to show representative tube‐like structure (total height 22.3 µm, z‐step 0.69 µm; right); H) Functional assessment of vessel‐like structures. FITC‐dextran molecules were added to the culture 1 day before fixation. The next day samples were washed, fixed and prepared for staining for immunofluorescence. Some FITC signal (depicted in red) overlapped with CD31‐positive structures (green); z‐stack projection total height 62.4 µm, z‐step 2.4 µm.

The lymphatic vessels formed by the co‐encapsulation of 2×10^6^ cells/mL of both hLECs and hBM‐MSCs within 1.7% TG‐PEG matrices were further characterized. The samples were supplemented with FGF‐2, VEGF‐A and VEGF‐C and cultured for 3 days. As evident from Figure 3A,B, the structures spanned approximately 250 µm in height and consisted of tube‐like hLEC structures accompanied by supporting cells, visualized by F‐actin staining. Staining for perivascular cell markers NG2 and PDGFRβ suggested that the supporting hBM‐MSCs were in contact with the hLEC network (Figure 3C). It has been found that PDGF‐B is essential for the recruitment of smooth muscle cells (SMCs) to lymphatic vessels, indicating that its receptor PDGFR is crucial for the structural integrity and functionality of collecting lymphatic vessels [39]. Moreover, the lymphatic structures were surrounded by the basement membrane components laminin and collagen IV (Figure 3D). This is in contrast to native lymphatic capillaries, where the basement membrane is typically present discontinuously [12, 40]. Upon closer investigation of the lymphatic structures, we could observe vascular endothelial (VE)‐cadherin staining, suggesting individual ECs formed cell‐cell junctions. An oak leaf‐like shape of endothelial cells was not observed [11], which would be expected in perfused native lymphatic capillaries [7, 41]. Nevertheless, the formed lymphatic networks retained the expression of lymphatic markers during the three‐day cultures. The lymphatic capillary marker LYVE‐1 was observed on some, but not all CD31‐positive cells (Figure 3F) and most nuclei of CD31‐positive cells stained positively for Prox‐1 (Figure 3G). The functionality of the CD31‐positive vessel‐like structures could not be confirmed, however, uptake of FITC‐labelled dextran molecules could be observed (Figure 3H). Prior to 3D culture, all cells stained positive for CD31 and Prox‐1, but only a fraction stained positively for LYVE‐1 (Figure S1B). The partial loss of the lymphatic marker LYVE‐1 occurred during passages in 2D culture (Figure S1B). We postulate that in this model, hLECs with a capillary‐specific phenotype might establish even more native lymphatic properties. In summary, our engineered lymphatic structures contain hallmarks of small native lymph vessels with a diameter of around 8 µm. Their association with perivascular support cells (staining positively for PDGFR) and the only partial staining for LYVE‐1 suggest that the formed structures resemble an intermediary of collecting vessels and lymphatic capillaries.

### 2.4 Lymphatic Vessels form in the Presence of Low Numbers of Cancer Cells

**FIGURE 4:**
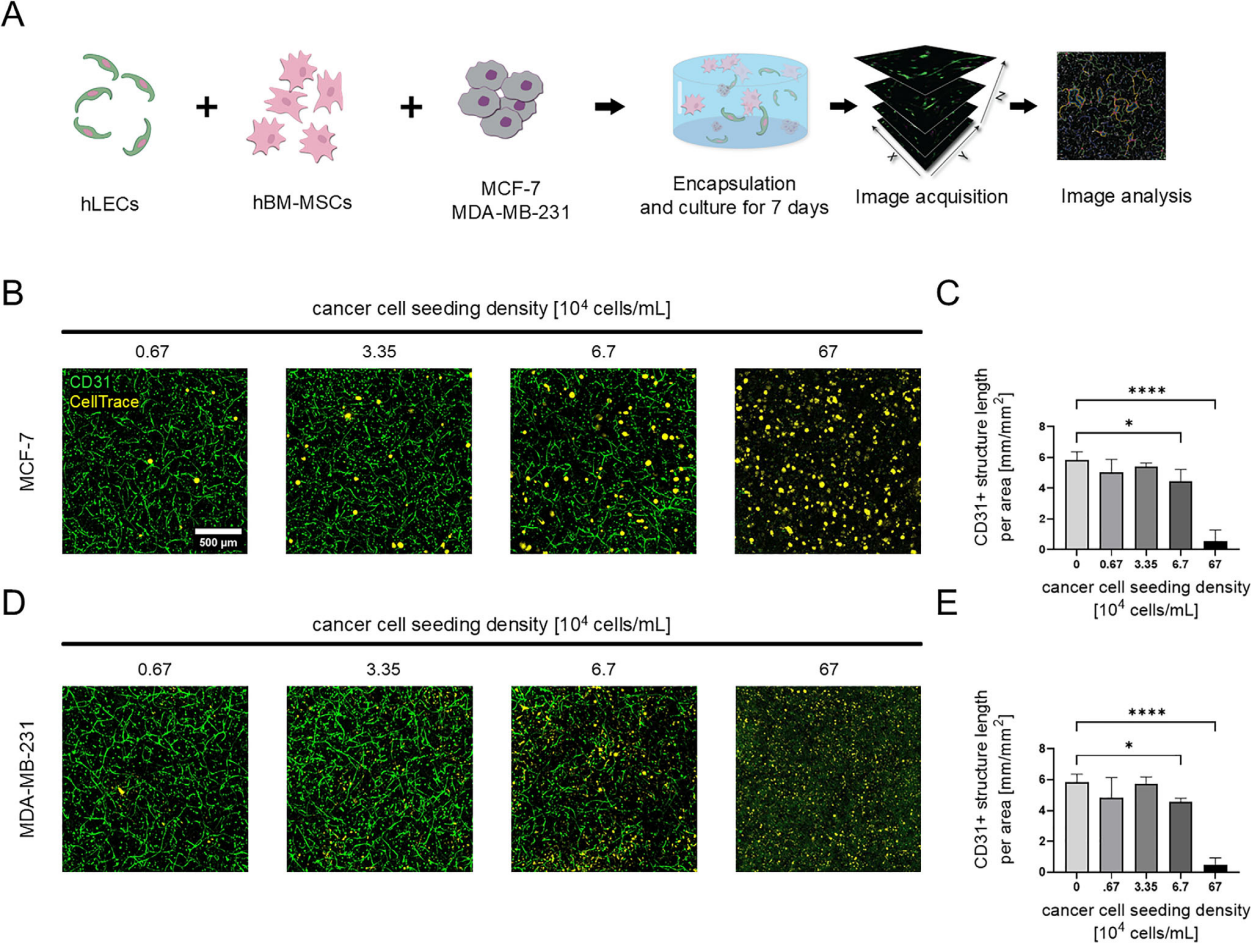
Cancer cells negatively influence growth of hLEC tube‐like structures. A) Schematic of the co‐encapsulation of hLECs, hBM‐MSCs and cancer cells and their consequent network quantification; B) MCF‐7 cancer cells were co‐encapsulated at varying concentrations, while hLEC and hBM‐MSC concentrations were kept at 2×10^6^ cells /mL. Representative images of confocal maximum intensity projections show distinct growth of cancer spheroids showing CD31‐positive structures in green and CellTrace‐labeled cancer cells in yellow; C) Quantification of the CD31‐positive signal revealed a significant length reduction in the presence of MCF‐7 cells at high concentrations (Ordinary one‐way ANOVA with Dunnett's Test *p < 0.05, ****p < 0.0001); D) Representative images of confocal maximum intensity projections show distinct mesenchymal spreading of cancer cells; E) Quantification of the CD31‐positive signal revealed a significant length reduction in the presence of MDA‐MB‐231 cells at high concentrations (Ordinary one‐way ANOVA with Dunnett's Test *p < 0.05, ****p < 0.0001).

The loose cell‐to‐cell contacts and discontinuous basement membrane likely render lymphatic capillaries the easiest entry point of metastasizing cancer cells into the lymphatic system [42, 43]. To recreate a tumor microenvironment in vitro, lymphatic networks were formed in the presence of the breast cancer cell lines MCF‐7 or MD‐MB‐231. Lymphatic networks were formed in TG‐PEG hydrogels using the lymphatic seeding conditions identified above. Co‐cultures of hLEC and hBM‐MSC at 2×10^6^ cells/ml each were encapsulated with different densities of CellTrace‐labelled cancer cells (0, 0.67×10^4^, 3.35×10^4^, 6.7×10^4^ and 67×10^4^ cancer cells/mL). After 7 days in culture in basal medium supplemented with 50 ng/mL FGF‐2, VEGF‐A and VEGF‐C, the constructs were fixed and prepared for image‐based network quantification (Figure 4A). At low numbers of cancer cells (0.67×10^4^, 3.35×10^4^ cancer cells/mL), lymphatic structures readily formed (Figure 4B, D). Here, the presence of cancer cells did not significantly affect CD31 network length (Figure 4C, E). However, with increasing numbers of cancer cells, the network formation was heavily stunted. For 6.7×10^4^ cancer cells/mL, networks still formed but to a lesser extent, whereas in the case of 67 × 10^4^ cancer cells/mL, networks were no longer able to form (Figure 4B–E). The inhibitory effect the two breast cancer lines had on the lymphatic vessel formation is in agreement with previous findings where lymphatic network growth was lower when MDA‐MB‐231 cells were present [22]. Interestingly, BEC network length was not significantly affected in co‐cultures with low numbers of cancer cells but with high numbers of cancer cells, network formation was also hindered (Figure S4B). Recent observations showed that lymphatic vessels are present in breast cancer and their density is increased primarily in the peri‐tumor area due to cancer‐initiated release of lymphangiogenic factors, such as VEGF‐C and VEGF‐D, VEGF‐A, or FGF‐2 [42]. These factors are secreted from various cells (leukocytes, macrophages and stromal cells) of the tumor microenvironment (TME) and might be further regulated by hypoxia [44, 45]. However, when inducing a hypoxic environment by adding 100 mM CoCl_2_ to the culture medium, CD31‐positive structures were not significantly affected (Figure S5B). Furthermore, qualitative assessment of staining for HIF‐1‐alpha, a known hypoxia marker, revealed that mostly cancer cells, rather than lymphatic cells, seemed to be in a hypoxic state (Figure S5C) [46]. Finally, since the formation of lymphatic vessel structures was not affected by the addition of cancer cells at lower densities, this model can be employed to study interactions between cancer cells and lymphatic vessels.

### 2.5 Invasive Breast Cancer Preferentially Interacts With Lymphatics

**FIGURE 5:**
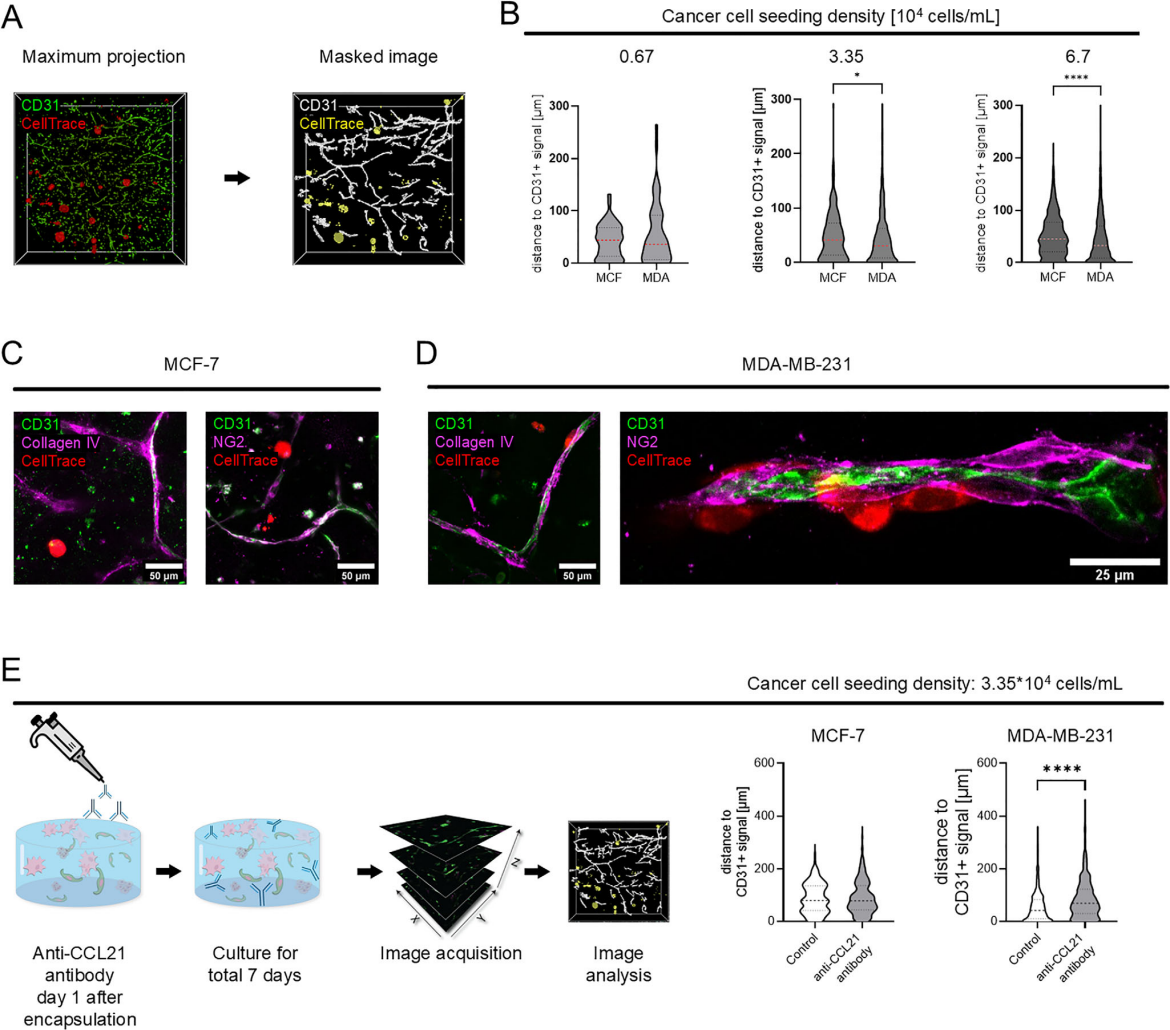
MDA‐MB‐231 cells show prioritized co‐localization to CD31‐positive tube‐like structures compared to MCF‐7. However, this co‐localization profile could be inhibited. A) Representative images to illustrate image processing for co‐localization analysis (CD31 signal in green, CellTrace signal in red); B) Violin plots of distance values of cancer cells (or cancer cell clusters) from the nearest CD31‐positive tube‐like structure, as segmented in A (MCF‐7: 0.67 ×10^4^ cancer cells/mL n = 55, 3.35×10^4^ cancer cells/mL n = 275, 6.7×10^4^ cancer cells/mL n = 1003) (MDA‐MB‐231: 0.67 ×10^4^ cancer cells/mL n = 50, 3.35×10^4^ cancer cells/mL n = 484, 6.7×10^4^ cancer cells/mL n = 1514) Mann‐Whitney test, *p < 0.05, ****p < 0.0001; C,D) Representative images of confocal maximum projections. CD31‐positive structures are depicted in green, collagen IV is depicted in magenta, perivascular cell marker NG2 is depicted in magenta and cancer cells stained with CellTrace are depicted in red. Left‐hand side images show MCF‐7 co‐cultures and right‐hand side images show MDA‐MB‐231 co‐cultures; E) Blocking of CCL21 inhibits association of MDA‐MB‐231 to lymphatic structures. The anti‐CCL21 antibody was added 1 day after the cells had been encapsulated. After a 7‐day culture, the samples were fixed and stained for image acquisition and analysis. Co‐localization measurement revealed a highly significantly reduced association of MDA‐MB‐231 to lymphatic structures when the anti‐CCL21 antibody was added (Mann‐Whitney test, ****p < 0.0001). No significant changes in migratory behavior were recorded in MCF‐7.

Lymphatic vessels can potentially direct the migration of cancer cells to peripheral lymphatic vessels by the release of chemokines such as C‐C motif chemokine Ligand 21 (CCL21), which binds to C‐C chemokine receptor type 7 (CCR7) present on both breast cancer cell lines MCF‐7 and MDA‐MB‐231 (Figure S6A) and might further influence their adhesion and invasion by the deposition of typical ECM components [47, 48, 49]. Therefore, in a next step, the relative localization of cancer cells to the lymphatic vascular structures was assessed. Z‐stacks were acquired using an automated imager and the signals for lymphatic structures (CD31; green) and cancer cells (CellTrace; red) were segmented in 3D (Figure 5A; example shown for MCF‐7). Next, the smallest distance between cancer cells and lymphatic structures was quantified for the cancer cell concentrations that did not totally impair lymphatic structure formation (i.e., 0.67×10^4^, 3.35×10^4^ and 6.7×10^4^ cancer cells/mL). As can be seen in the plots in Figure 5B, MCF‐7 clusters localized at a median distance of 44 µm (0.67×10^4^ cancer cells/mL n = 55 cancer clusters), 41 µm (3.35×10^4^ cancer cells/mL n = 275 cancer clusters) and 45 µm (6.7×10^4^ cancer cells/mL n = 1003 cancer clusters). In contrast, MDA‐MB‐231 appeared to be in closer contact with the lymphatic structures at a median distance of 36.0 µm (0.67×10^4^ cancer cells/mL n = 50 single cancer cells), 31 µm (3.35×10^4^ cancer cells/mL n = 484 single cancer cells) and 34 µm (6.7×10^4^ cancer cells/mL n = 1514 single cancer cells). This analysis confirms an empirical trend observed while culturing and imaging. Representative images are shown in Figure 5C. While it was rare to find MCF‐7 clusters directly in contact with vessel‐like structures, single MDA‐MB‐231 cells could often be seen adhering to the structures, or, more specifically, to the supporting cells. This contrast may be due to the inherent differences between the two cancer cell lines and their associated migratory (or non‐migratory) behavior [50]. Therefore, in a next step we aimed to target this migratory behavior. The encapsulated co‐cultures were maintained in basal medium supplemented with 50 ng/mL FGF‐2, VEGF‐A and VEGF‐C. After day 1 the medium was exchanged and the treatment group was also supplemented with the anti‐CCL21 blocking antibody. Interestingly, by adding an anti‐CCL21 blocking antibody to the cultures, significantly fewer MDA‐MB‐231 cells were in close association with lymphatic structures (Figure 5E). In fact, the median distance from lymphatic structures increased from 42 µm of no antibody treatment to 69 µm with antibody treatment. No distance changes were recorded in MCF‐7 co‐cultures (78 µm when untreated and 79 µm with anti CCL21 treatment). This further supports the hypothesis that lymphatic vessels may direct the migration of cancer cells by the release of chemokines such as CCL21.

## 3 Conclusions

In this study, we established a 3D lymphatic vessel model in a fully defined biomimetic hydrogel. We hypothesize that this model can be used to study the interaction of cancer cells with lymphatic vessels. So far, only a limited number of lymphatic vessel models have been described. Many of these previous models were formed in naturally occurring matrices, such as collagen scaffolds or fibrin, showing promising functionality of the lymphatics [20, 21, 22]. However, they are limited in the flexibility of independently engineering material parameters, such as matrix stiffness and stability, and controlled presentation of biological signals. In the synthetic matrix used in this study, supporting cells modulate the initial substrate by depositing cell‐inherent ECM components. This minimalistic environment, where cells are their own architects results in the formation of tissue constructs. These constructs at least partially represent the properties of the microenvironment cells are exposed to in the native situation. The model presented here provides a 3D environment where specific cellular interactions between hLECs, supporting cells and cancer cells can be investigated in the presence or absence of lymphangiogenic conditions. Importantly, in this experimental setup the invasive MDA‐MB‐231 and the non‐invasive MCF‐7 cancer cell lines showed significantly different growth and spreading behaviors. The close interaction of the MDA‐MB‐231 cells with the lymphatic structures might reflect their invasive potential and could serve to investigate mechanisms that contribute to invasiveness. Importantly, by blocking CCL21 activity, the close association of MDA‐MB‐231 with the engineered lymphatic structures was inhibited. Future studies may be conducted in synthetic hydrogels with an interconnected microporosity to facilitate the formation of a continuous and perfusable lumen. This could allow for more functional evaluations of the interactions of different cancer cells with the lymphatic vasculature or for the study of lymphangiogenesis.

## 4 Experimental Section/Methods

### 4.1 Cell Culture

Human lymphatic endothelial cells (hLECs; Lonza CC‐2812, lot: 209333;) were grown in EBM‐2 (Lonza) supplemented with EGM‐2 SingleQuots (Lonza) and 10% FBS (Gibco). Human dermal lymphatic endothelial cells (hDLEC, Promocell C‐12217, lot: 489Z014) were grown in Endothelial Cell Growth Medium MV2 Kit (Promocell). Passages 5–11 were used. hLEC/hDLEC characterization was confirmed via positive expression of pan‐endothelial marker CD31, lymphatic specific Prox‐1, LYVE‐1 and podoplanin (Figure S1B). For assessments of blood endothelial cells (BECs), GFP‐expressing human umbilical vein endothelial cells (GFP‐HUVECs) were used. GFP‐HUVECs purchased from ABC Biopply were cultured in EBM‐2 supplemented with EGM‐2 SingleQuots (Lonza) and 10% FBS (Gibco). Passages 6–11 were used. Endothelial cells were cultured in tissue culture flasks (TPP) coated with collagen I (150 µg/mL, Corning, rat‐tail). Human bone marrow‐derived mesenchymal stromal cells (hBM‐MSCs) were provided by the University Hospital Basel and isolated as described previously [51]. In accordance with the local ethical committee (University Hospital Basel; Prof. Dr. Kummer; approval date 26/03/2007 Ref Number 78/07), patients undergoing orthopedic surgical operations gave consent to harvest bone tissue and the donors were anonymized. hBM‐MSCs were cultured in MEM alpha (Gibco) supplemented with 10% FBS (Gibco), 1% penicillin‐streptomycin (Pen‐Strep, Gibco) and 5 ng/mL human recombinant FGF‐2 (Peprotech). Passages 5–10 were used. Human breast cancer cell lines (MCF‐7, ATCC, RRID:CVCL_0031) and MDA‐MB‐231 (ATCC, RRID:CVCL_0062) were kindly obtained from J. Massagué at the Memorial Sloan‐Kettering Cancer Center) were maintained in DMEM/F12 (Gibco) supplemented with 10% FBS and 1% Pen‐Strep. All cells were cultured at 37°C 5% CO2 in a humidified atmosphere. All cells tested negatively to contamination from mycoplasma.

### 4.2 TG‐PEG Hydrogel Formation and Culture

TG‐PEG hydrogel precursors were synthesized as previously described [28]. In short, 8‐arm polyethylene glycol (PEG) polymers were functionalized with peptides, namely glutamine and lysine. The lysine sites included a matrix metalloproteinase (MMP) cleavable site to render the TG‐PEG hydrogels enzymatically degradable. Finally, the PEG polymers with molecular length of 40 kDa were prepared in Tris buffer (50 mM, pH 7.6). Cells were added at the specified concentrations before crosslinking. TG‐PEG polymers were crosslinked by adding 10 U/mL coagulation factor XIIIa (a transglutaminase or TG), which was previously prepared by activating factor XIII (Fibrogammin, Behring) with thrombin (0.02 U/µL, Sigma) for 30 min at 37°C. CaCl2 (50 mM) was added as co‐factor for the crosslinking reaction of TG‐PEG. Disk‐shaped hydrogels of 15 uL were polymerized between two glass slides which had been hydrophobically treated with SigmaCote (Sigma‐Aldrich), separated by 1.00 mm spacers, resulting in disk‐shaped hydrogels of 1 mm height and 4.4 mm diameter. Finally, hydrogels were cultured in MEM alpha supplemented with 10% FBS, 1% Pen‐Strep and growth factors (human recombinant FGF‐2, VEGF‐A 165, or VEGF‐C concentrations of 50 ng/mL; all from Peprotech). Medium was replaced every 3–4 days

### 4.3 RNA Extraction, cDNA Synthesis, Reverse Transcriptase Polymerase Chain Reaction (RT‐PCR), PCR Product Visualization

Total RNA was extracted from cultured cells using the RNeasy Microkit (Qiagen) following manufacturer's instructions. cDNA was synthesized following the instructions of the High Capacity cDNA Reverse Transcription kit (Applied Biosystems). Per cell‐line 0.14 µg were used. Primers for human CCR7 and GAPDH were synthesized at Microsynth (Primer sequences Fig S6B). PCR was performed with Q5 Hot Start High‐Fidelity 2X Master Mix (New England Biolabs). PCR products were visualized via agarose gel electrophoresis (Agarose NEEO Ultra‐Quality, Carl Roth GmbH, Charge 510304406). 1% agarose gels were run and then imaged (Fusion FX, Vilber).

### 4.4 CCL21 Blocking

hLECs, hBM‐MSCs and cancer cells were co‐encapsulated at the specified concentrations. After culture for 1 day in culture medium (MEM alpha supplemented with 10% FBS, 1% Pen‐Strep and growth factors) the medium was changed to culture medium with 0 and 4 µg/mL human CCL21/6Ckine antibody (R&D Systems AF366‐SP) After 7 days, the samples were fixed and prepared for image analysis.

### 4.5 Hypoxia Environment

CoCl2 was used to induce a hypoxic environment. Before culturing with CoCl2, viability of the cells to CoCl2 was assessed (Figure S5A). For this, the cell types were cultured in 48 well plates and CoCl2 was added at concentrations of 0, 10, 100, and 1000 µM. After 24 h incubation the cultures were stained with LIVE/DEAD Viability/Cytotoxiticy Kit (Invitrogen). After incubation of 30 min the samples were imaged. Whole‐well images were processed with a median blur and thresholded. Quantification of live and dead signal was acquired using particle counter in Fiji. Co‐cultures consisting of hLECs, BM‐MSCs and cancer cells were encapsulated and after 3 days of culture treated with culture medium supplemented with 100 µM CoCl2 or H2O control. After 24 h incubation with the hypoxic agent the medium was changed and after a total of 7 days the samples were fixed and processed for image‐based analysis.

### 4.6 In Situ Rheology

An Anton Paar MCR 302e rheometer was used for rheological analysis of 12 µL cell‐free TG‐PEG hydrogels (1.3%, 1.7%, and 2.1% w/v). An 8 mm parallel plate geometry (PP8, Anton Paar) was used. Tests to characterize crosslinking dynamics of TG‐PEG hydrogels were performed at 37°C. To prevent drying, silicone oil was applied around the polymerizing gel. The gap size was set to 200 µm and measurements were recorded every 10 s for 30 min. Oscillatory measurements were set to a shear strain of 4% and 1 Hz frequency.

### 4.7 Fixation and Staining

Before fixation, the 15 µL gels were washed with sterile PBS. Hydrogels were then fixed for 30 min at room temperature in 4% paraformaldehyde in PBS (Artechemis). The hydrogels were permeabilized in 0.3% Triton X‐100 (AppliChem) with simultaneous blocking using 1% BSA (AppliChem) in PBS at room temperature for 30 min. For primary and secondary staining, the hydrogels were incubated overnight at 4°C. Primary staining of extracellular matrix, such as laminin or collagen IV, was performed while the samples remained in culture, with primary antibody incubation in culture medium for 8–12 h before fixation. Between every step of fixation, permeabilization, and staining, the hydrogels were washed with PBS.

### 4.8 Functional Assays

Dextran uptake for measuring capillary infiltration was performed following previously published protocols [52]. In short, 1 day before fixation the samples were incubated in culture medium with FITC‐labeled dextran molecules (0.1 mg/mL, MW 20 kDa, Sigma Lot SLBH1156V). The next day the samples were washed, fixed and stained for CD31, DAPI and F‐actin.

### 4.9 Image Acquisition

Fixed and immunostained samples were imaged with a Leica Stellaris 5 inverse confocal laser scanning microscope (CLSM). The HC PL FLUOTAR 5x objective was used with 0.75x digital zoom to acquire whole‐gel images. Whole‐gel confocal images were acquired as a z‐stack, with a stack height 220 µm and step size 10 µm. Close‐up images were acquired with the HC PL APO CS2 10x and 20x objective with digital zoom ranging from 1x to 10x. For co‐localization studies, the Operetta CLS (PerkinElmer) high throughput microplate imager was used. Whole gel stacks were acquired with the 10x objective.

### 4.10 Image Processing

The CD31‐positive signal was analyzed using the Angiogenesis Analyzer for Fiji [35]. Maximum intensity projections of the whole‐gel z‐stacks were assessed. Before analysis, the signal was pre‐processed, adding a blur of radius 1.0‐2.0 and turning the color code from 8‐bit into RGB color. The readout of total length in pixel was converted to length in mm and divided by the total area measured in mm2. This ratio of the CD31‐positive signal was used for the analysis.

### 4.11 Co‐Localization

Whole gel stacks of both the CD31‐positive signal of the hLECs and the CellTrace signal of labelled cancer cells were acquired at the Operetta CLS. For the proximity analysis the bottom 150 µm were discarded. A mask was used to remove single CD31‐positive cells. 3D segmentation of CD31‐positive structures and the CellTrace signal was performed. Afterward, a mask for the CD31‐positive signal and for the CellTrace signal was created and the minimum distance of each cancer cell/cancer spheroid to the lymphatic structures were calculated using the Harmony software (workflow depicted in Figure S7).

### 4.12 3D Network Quantification

The Qiber3D Python package was used as previously described [53]. The images were preprocessed as follows: a 3 × 3 median filter was applied to reduce noise followed by a Hessian‐based filter. The images were binarized using an intensity threshold. Next, morphological dilation and Gaussian smoothing was performed and small island removal was set to a filtersize of 100 pixel. Afterwards, 3D reconstruction and morphometric measurements were done with the default settings.

### 4.13 Data Analysis

Statistical analysis was performed using Graphpad Prism (GraphPad software version 9.5.1, San Diego, CA, USA) and significance determined using one‐way ANOVA with a Tukey post‐hoc correction. Each experiment was performed at least two times and each experiment had n≥3 technical replicates, if not mentioned otherwise.

* indicates p‐value 0.01 to 0.05 (significant)

** indicates p‐value 0.001 to 0.01 (very significant)

*** indicates p < 0.001 (highly significant)

**** indicates p < 0.0001 (extremely significant)

### 4.14 Antibodies Used

The following primary antibodies were used for immunofluorescence staining: rabbit anti‐Prox‐1 (1:300, ReliaTech, 102‐PA32AG), rabbit anti‐LYVE‐1 (1:300, ReliaTech, 102‐PA50), mouse anti‐CD‐31 (1:300, BD Bioscience, BD555444), mouse anti‐podoplanin (1:200, Santa Cruz, sc‐376695), rabbit anti‐laminin (1:200, Abcam, ab7463), rabbit anti‐VE‐Cadherin (1:200, Abcam, ab33168), rabbit anti‐PDGFRβ (1:200, CellSignalling, 3169T), rabbit anti‐NG2 (1:200, Abcam, ab275024) and rabbit anti‐HIF‐1 α (1:300, R&D Systems, MAB19352). Afterward the samples were treated with the following secondary antibodies: AlexaFluor 488 goat anti‐mouse or goat anti‐rabbit (both 1:300, Abcam), DyLight 649 goat anti‐mouse or donkey anti‐rabbit (both 1:300, Biolegend), AlexaFluor 555 goat anti‐mouse (1:200, Biolegend) or donkey anti‐rabbit (1:200 Bioss). Additionally, these fluorescent stainings were used: DAPI (1 µg/mL, Sigma/Invitrogen), phalloidin‐rhodamine (1:4000, Sigma/Invitrogen), CellTrace Far Red (according to the manufacturer's instructions, Thermo Fisher).

### 4.15 Swelling Ratio Determination

Swelling ratios of TG‐PEG hydrogels were assessed for concentrations of 1.3%, 1.7%, and 2.1% PEG w/v. Cell‐free hydrogels of 15 µL were polymerized and immediately weighed. After weighing, the hydrogels were transferred into 24‐well plates and submerged in warm MEM alpha. After 24 h, the hydrogels were weighed again and the swelling ratio was calculated as the weight after 24 h divided by the initial weight directly after casting.

## Funding

This work was supported by Swiss National Science Foundation (SNSF) grants number 310030E_202429, 205321_204318, IZJFZ3_202474.

## Conflicts of Interest

The authors declare no conflict of interest.

## Supporting information




**Supporting File**: adhm70779‐sup‐0001‐SuppMat.docx.

## Data Availability

The data that support the findings of this study are available from the corresponding author upon reasonable request.
