# Supplementary material for: Bioengineered Lymphatic Vessels in Synthetic Matrices to Study Breast Cancer Cell Functions
Source: Adv Healthc Mater. 2026 Jan 21;15(13):e03325. doi: 10.1002/adhm.202503325 (PMC13058782; doi:10.1002/adhm.202503325)
Supplement: Supplementary file 1 — Supporting File: adhm70779‐sup‐0001‐SuppMat.docx. [file ADHM-15-0-s001.docx]

# Supporting Information

Bioengineered Lymphatic Vessels in Synthetic Matrices to Study Breast Cancer Cell Functions

*Rodi Odabasi, Lisa A. Krattiger, Magdalini Kanari, Mira A. Jacobs, Lukas O. Moser, Mark W. Tibbitt, Cornelia Halin and Martin Ehrbar*

**
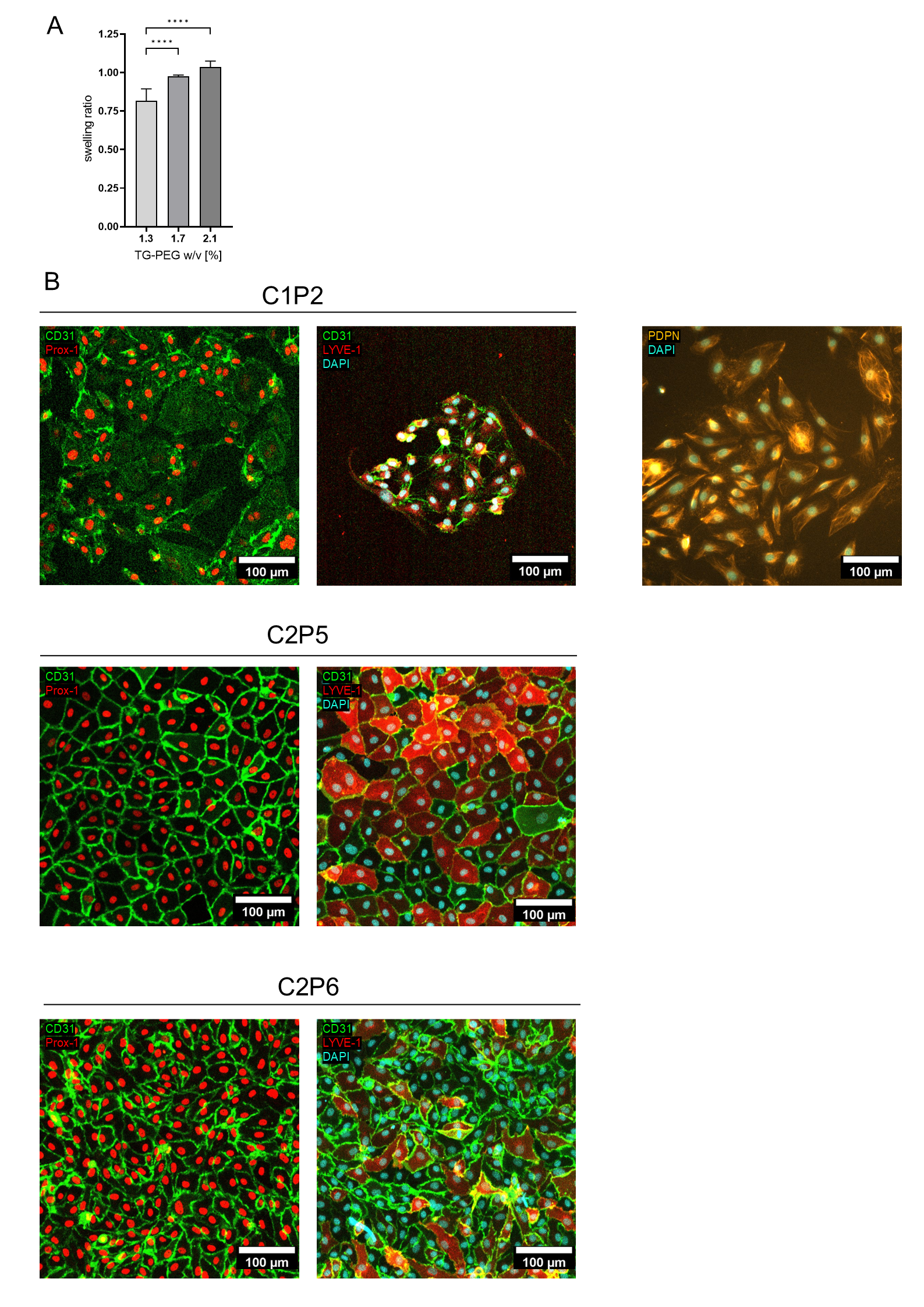
Figure S1**. Swelling properties of TG-PEG hydrogels and lymphatic markers during passage. A) Swelling ratio of TG-PEG matrices at different weight percentages. Quantification of the swelling ratio of 1.3, 1.7 and 2.1% w/v TG-PEG matrices as determined by weighing hydrogels before and after overnight incubation in culture medium. n≥7 technical replicates. Ordinary one-way ANOVA with Tukey-Kramer test; ****p < 0.0001; B) lymphatic specific markers Prox-1 (red), LYVE-1 (red) and podoplanin (yellow) of hLEC at different passages. Prox-1 expression stayed stable over passages, while LYVE-1-positive cells decreased with passages.


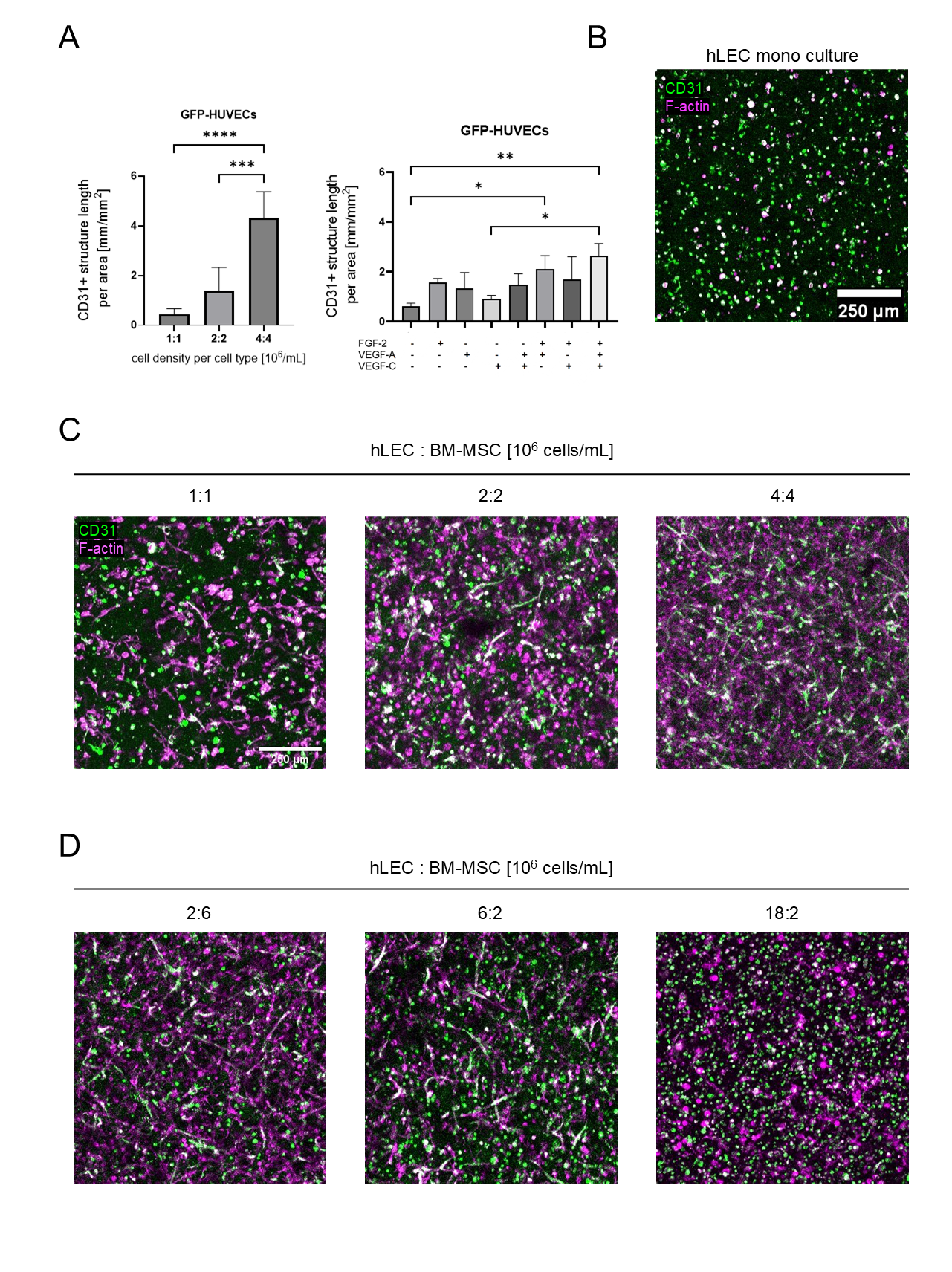


**Figure S2**. Assessment of BEC networks and qualitative co-staining of hLEC and BM-MSCs, A) Quantification of total GFP signal length, firstly different seeding densities were assessed (1×10^6^ cells/ml, 2 ×10^6^ cells/ml and 4 ×10^6^ cells/ml GFP-HUVECs and hBM-MSC co-cultures, n≥5). Higher cell concentrations yielded significantly higher network length. Next, different growth media were assessed, where supplementation with 50ng/mL of each FGF-2, VEGF-A and VEGF-C yielded highest network length (n≥3); B) hLEC monocultures remain round in TG-PEG matrices. Confocal maximum intensity projection (total height 210 µm, z-step 10 µm, CD31 signal in green, f-actin in magenta) of hLEC monocultures encapsulated at 2×10^6^ cells/mL within a 1.7% TG-PEG matrix. Cultures were kept for 3 days in the presence of 50 ng/mL FGF-2; C) Confocal maximum intensity projections (total height 210 µm, z-step 10 µm, CD31 signal in green, f-actin in magenta) of different cell densities (same regions as in Figure 2A). D) Confocal maximum intensity projections (total height 210 µm, z-step 10 µm) of different cell ratios (same regions as in Figure 2C).

**
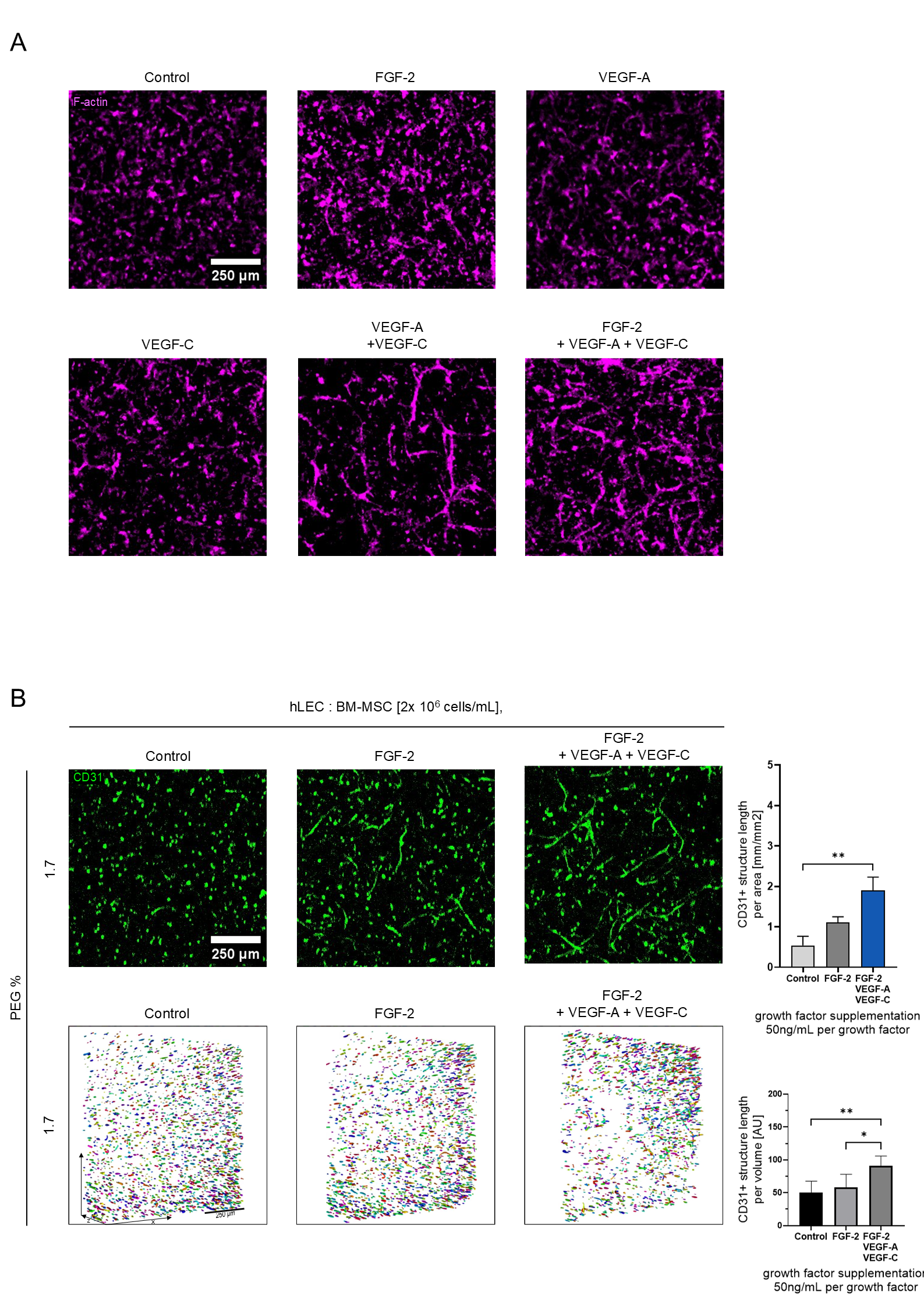
**

**Figure S3**: Qualitative assessment of supporting cells and assessment of juvenile hLEC source. A) Confocal maximum intensity projection (total height 210 µm, z-step 10 µm, f-actin in magenta) for qualitative assessment of different growth factor supplementation (same regions as in Figure 2E; B) Growth factor assessment with juvenile hLEC source: Representative confocal maximum projections (total height 210 µm, z-step 10 µm, CD31 in green) of different growth factor supplementations and their quantification (n≥3), significantly longer networks formed when supplemented with 50ng/mL of each FGF-2, VEGF-A and VEGF-C. The results were confirmed using a 3D quantification tool (Qiber3D).


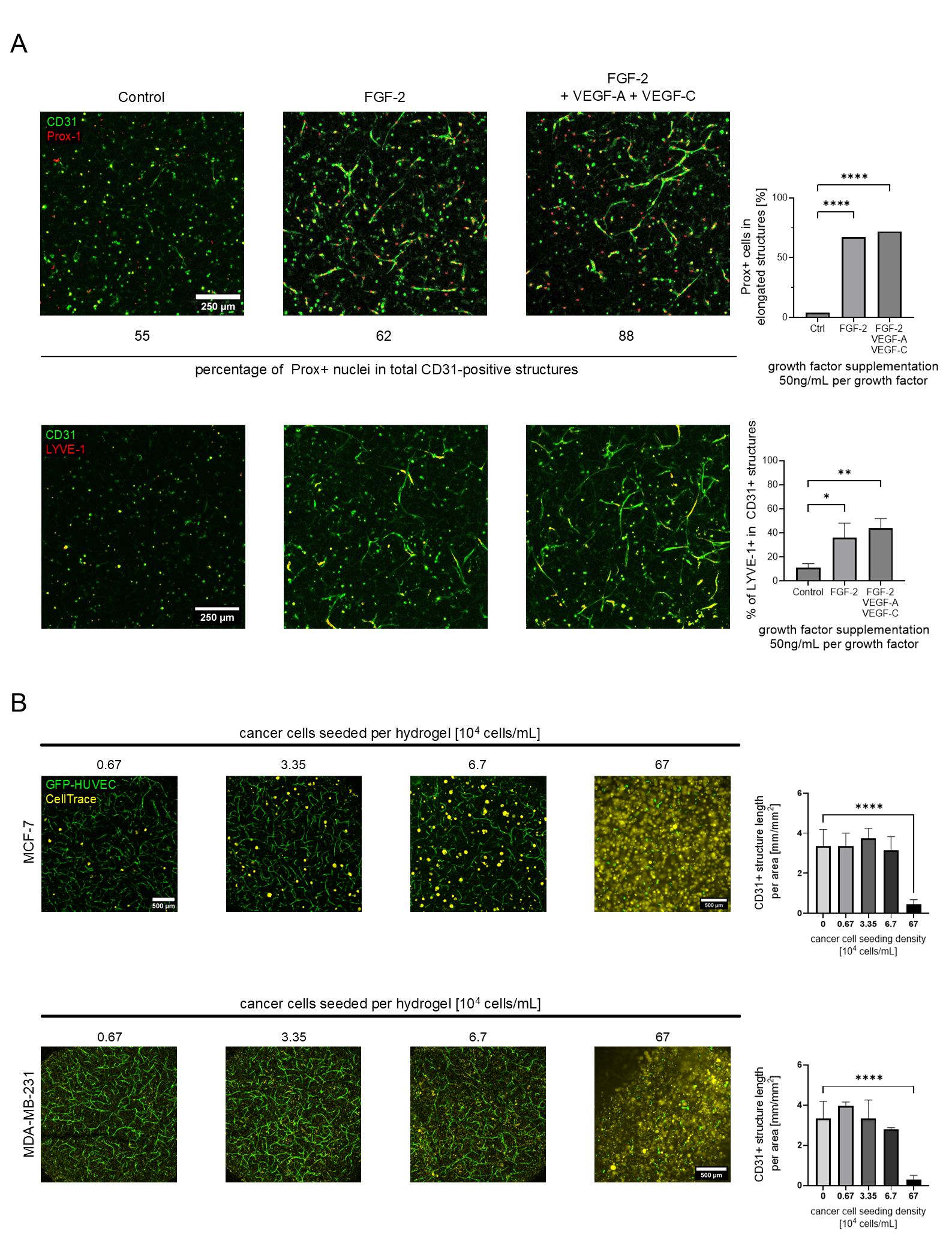


**Figure S4**: Assessment of culture conditions to lymphatic markers and assessment of BEC BM-MSCs in co-culture with cancer cells. A) Culture conditions affect quality of vessels. With growth factor supplementation more Prox-1-positive cells (depicted in red) are in elongated CD31-positive structures (depicted in green). More LYVE-1-positive structures (depicted in red) are present in elongated CD31-positive structures (depicted in green)when growth-factor is added; B) co-encapsulation of different cancer densities cell in GFP-HUVEC hBM-MSC co-cultures. Representative confocal images and quantified network lengths (total height 210 µm, z-step 10 µm, GFP signal in green, CellTrace in yellow). Addition of low to medium concentrations of cancer cells did not significantly alter vessel-length of GFP-HUVEC structures; However at high concentrations vessel formation was completely inhibited (n≥3).


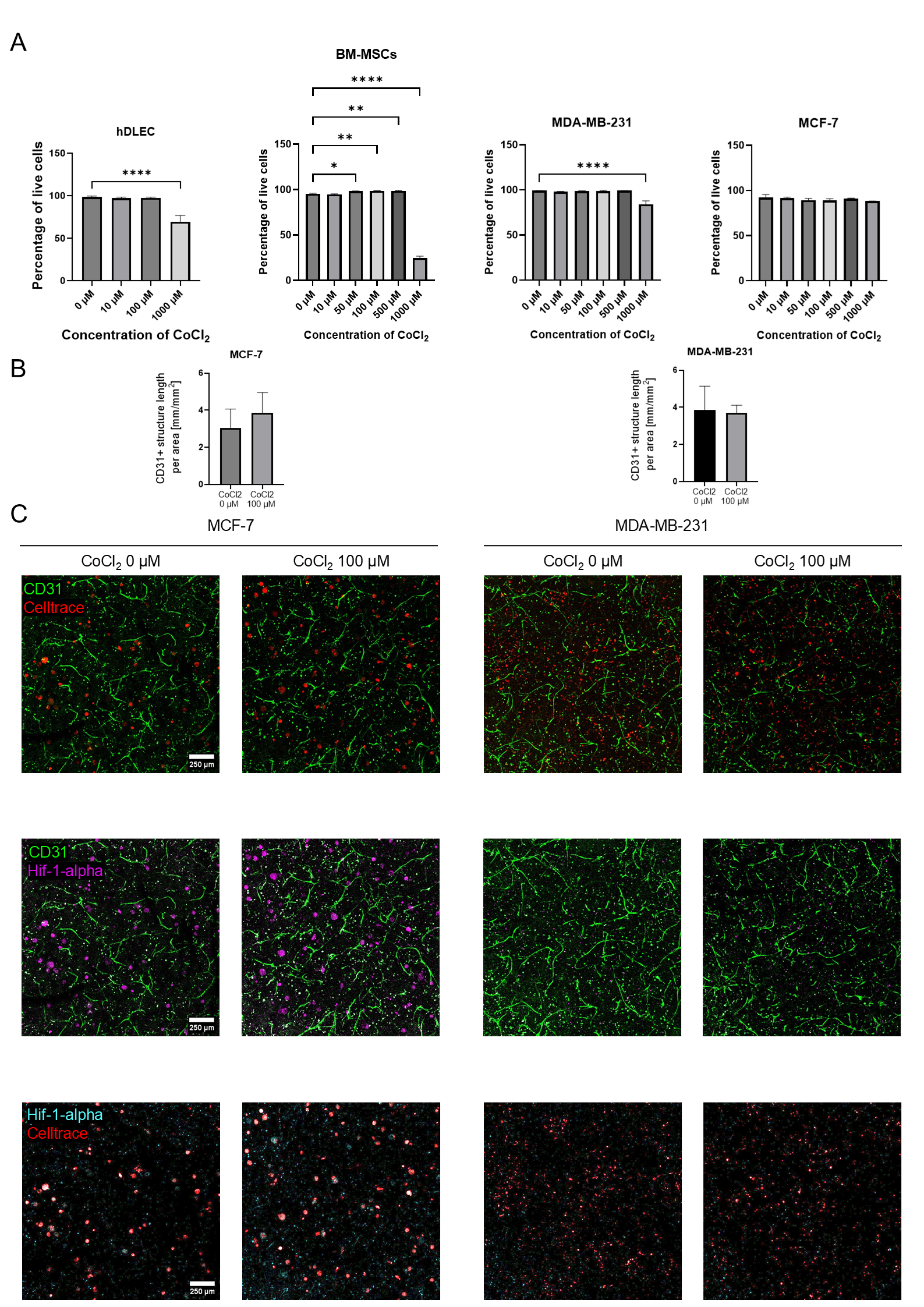


Figure S5: Effect of hypoxic conditions in 3D lymphatic cultures; A) Viability off involved cell types was assessed to increasing CoCl_2_ concentrations (incubation time 24h). Later co-cultures of hLECs, hBM-MSCs and cancer cells were incubated with 100 µM CoCl_2_ for 24h; B) No changes in vessel formation could be observed; C) Representative maximum projections of confocal images (total height 210 µm, z-step 10 µm, CD31 in green, CellTrace in red) staining for the hypoxic marker Hif-1-alpha (depicted in magenta and cyan) showed already a high number of hypoxic cells in the non-hypoxic condition.


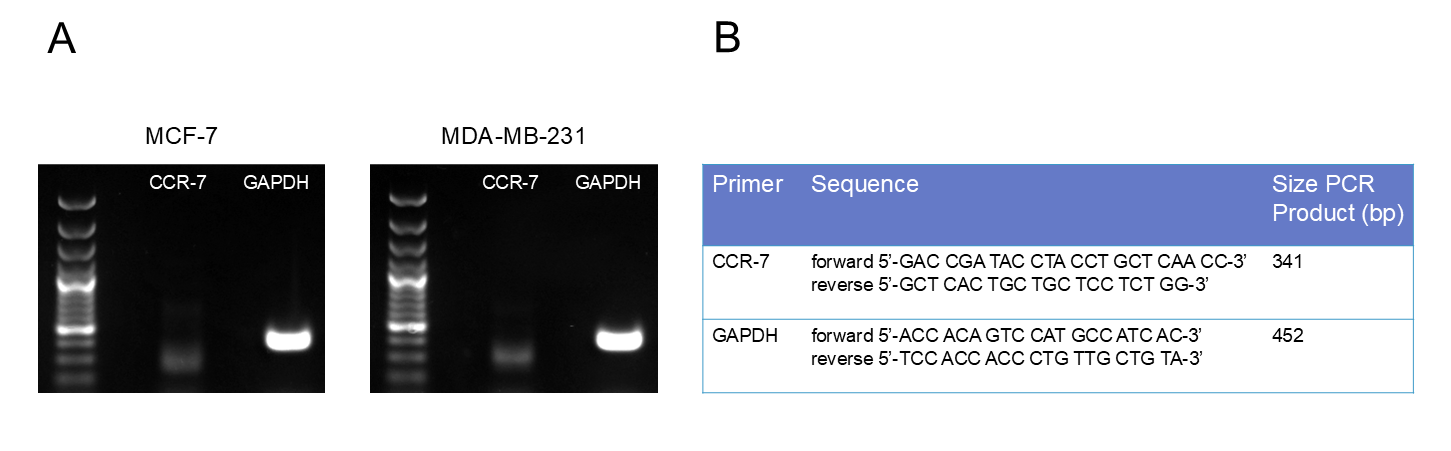


Figure S6: A) Assessment of CCR-7 expression in MCF-7 and MD-MB-231; B) Primer sequences for CCR-7 and GAPDH.


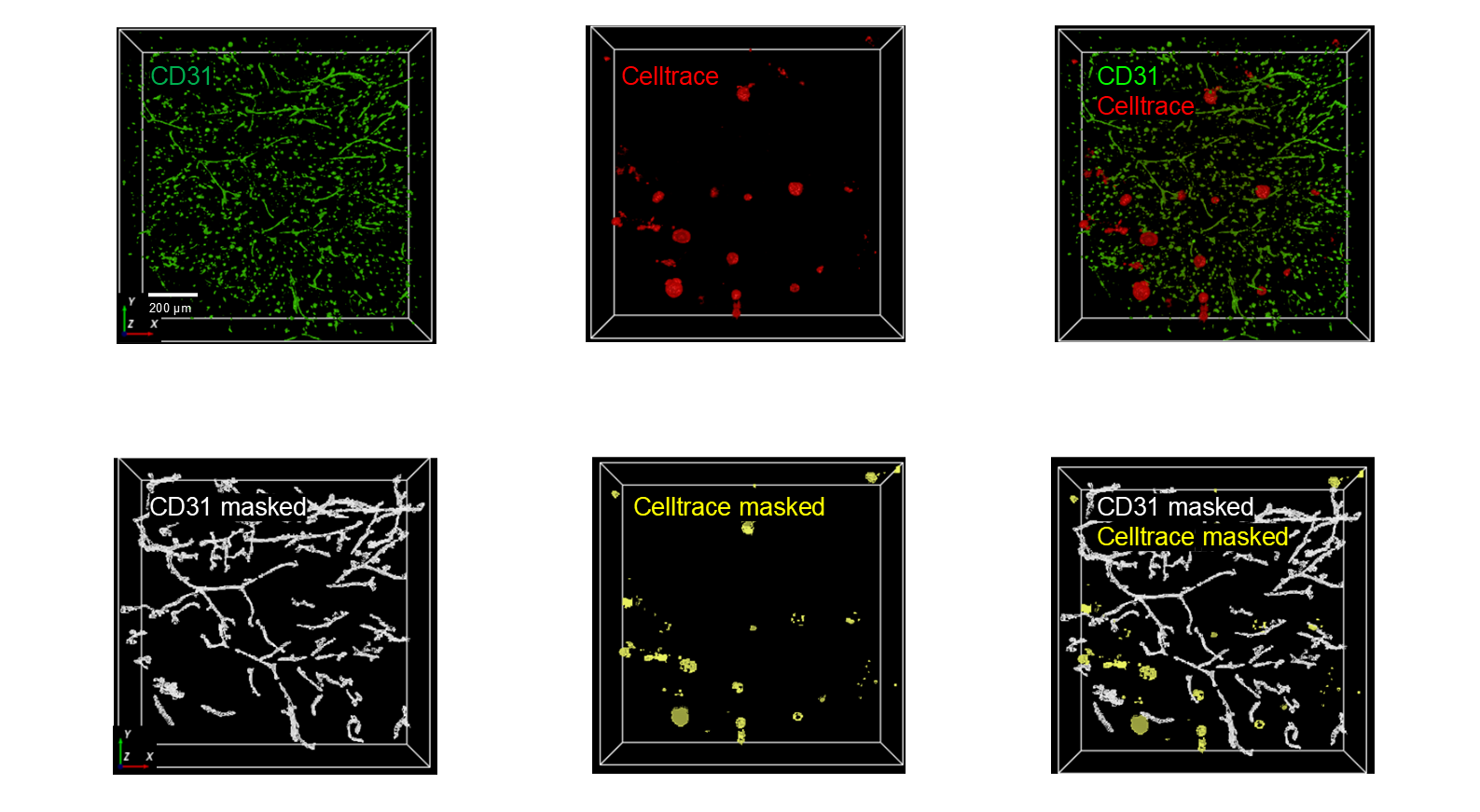


Figure S7: Workflow for operetta quantification: Images were acquired and a mask of the 3D reconstruction of the channels was created. Next, using the Harmony Operetta software, the minimum distance of each cancer cell/spheroid to the nearest CD31 structures was calculated.
